# The Potential Roles of Osmotic and Nonosmotic Sodium Handling in Mediating the Effects of Sodium-Glucose Cotransporter 2 Inhibitors on Heart Failure

**DOI:** 10.1016/j.cardfail.2021.07.003

**Published:** 2021-07-18

**Authors:** PETTER BJORNSTAD, PETER J. GREASLEY, DAVID C. WHEELER, GLENN M. CHERTOW, ANNA MARIA LANGKILDE, HIDDO J.L. HEERSPINK, DANIËL H. VAN RAALTE

**Affiliations:** 1 University of Colorado Anschutz Medical Campus and Children’s Hospital Colorado, Aurora, Colorado; 2 BioPharmaceuticals R&D, AstraZeneca, Gothenburg, Sweden; 3 Department of Renal Medicine, University College London, London, UK; 4 The George Institute for Global Health, Sydney, Australia; 5 Division of Nephrology, Department of Medicine, Stanford University School of Medicine, Palo Alto, California; 6 Department of Clinical Pharmacy and Pharmacology, University of Groningen, University Medical Center Groningen, Groningen, the Netherlands; 7 Diabetes Center, Department of Internal Medicine, Amsterdam University Medical Center, location VUMC, Amsterdam, the Netherlands

**Keywords:** Heart failure, natriuresis, nonosmotic sodium, SGLT2 inhibitors

## Abstract

Concomitant type 2 diabetes and chronic kidney disease increases the risk of heart failure. Recent studies demonstrate beneficial effects of sodium-glucose cotransporter 2 (SGLT2) inhibitors on chronic kidney disease progression and heart failure hospitalization in patients with and without diabetes. In addition to inhibiting glucose reabsorption, SGLT2 inhibitors decrease proximal tubular sodium reabsorption, possibly leading to transient natriuresis. We review the hypothesis that SGLT2 inhibitor’s natriuretic and osmotic diuretic effects mediate their cardioprotective effects. The degree to which these benefits are related to changes in sodium, independent of the kidney, is currently unknown. Aside from effects on osmotically active sodium, we explore the intriguing possibility that SGLT2 inhibitors could also modulate nonosmotic sodium storage. This alternative hypothesis is based on emerging literature that challenges the traditional 2-compartment model of sodium balance to provide support for a 3-compartment model that includes the binding of sodium to glycosaminoglycans, such as those in muscles and skin. This recent research on nonosmotic sodium storage, as well as direct cardiac effects of SGLT2 inhibitors, provides possibilities for other ways in which SGLT2 inhibitors might mitigate heart failure risk. Overall, we review the effects of SGLT2 inhibitors on sodium balance and sensitivity, cardiac tissue, interstitial fluid and plasma volume, and nonosmotic sodium storage.

## Lay summary

SGLT2 inhibitors have cardiovascular benefits that include HF outcomes in patients with and without diabetes. Because the underlying mechanisms are only partly explained by improvements in BP, body weight, or glucose control, other mechanisms have been proposed. We focus here on a central role for effects on sodium as underlying the positive benefits of SGLT2 inhibitors in HF. We explore the new (although still unconfirmed) idea that SGLT2 inhibitors exert some of their positive effects by affecting nonosmotic sodium (ie, sodium bound to muscles and skin and not dissolved in the blood).

- SGLT2 inhibitors have emerged as a class of drugs, previously prescribed for patients with T2D, that have in more recent years been shown to have substantial heart and kidney clinical benefits in patients with and without T2D.
- The degree to which these benefits are related to kidney-independent changes in sodium homeostasis is currently unknown.
- A better understanding of the nonosmotic mechanisms underpinning the benefits of SGLT2 inhibition on HF (with reduced or preserved left ventricular ejection fraction) may allow researchers to assess the effects of SGLT2 inhibitors in combination with other treatments that affect sodium balance.

Type 2 diabetes (T2D) is an established risk factor for ischemic cardiovascular disease (CVD) and heart failure (HF).^1^ The risks of ischemic CVD and HF are increased with albuminuria and/or impaired kidney function. Although in recent decades cardiovascular outcomes have improved for adults with or without T2D, decreasing the burden associated with HF by treating classical cardiovascular risk factors has proven to be difficult and thus remains a major public health priority.^2^ Accordingly, the introduction of sodium-glucose cotransporter 2 (SGLT2) inhibitors offers promise to mitigate cardiorenal disease in people with or without T2D. However, to better understand the role of these drugs in the cardiovascular system, it is important to define their mechanism of action on the cardiorenal axis.

The kidney contributes to glucose homeostasis by actively reabsorbing nearly all of the filtered glucose in the proximal tubule. Although the kinetics of renal glucose reabsorption were first described nearly 90 years ago, it took until the early 1970s to demonstrate that glucose reabsorption occurs in the proximal tubule through 2 distinct sodium-glucose cotransport systems. Shortly thereafter, 2 SGLTs (SGLT1 and SGLT2) were discovered.^3^ SGLT1 is a high-affinity, low-capacity transporter responsible for approximately 10% of the renal glucose reabsorption; SGLT2 is a low-affinity, high-capacity transporter responsible for approximately 90% of the renal glucose reabsorption.^4,5^ Together, these transporters are thought to be responsible for total renal glucose reabsorption. In addition to glucose reabsorption, SGLT1 and SGLT2 also facilitate concomitant sodium reabsorption. Approximately two-thirds of the total kidney sodium reabsorption occurs in the proximal tubule, although the extent to which this reabsorption is mediated by SGLT1 and SGLT2 presently remains unknown.^6^

SGLT2 inhibitors were granted marketing authorization in 2014 as glucose-lowering drugs, and work by inducing glucosuria. Through their mechanism of action, the glucose-lowering effects of SGLT2 inhibitors in patients with chronic kidney disease (CKD) are modest.^7^ However, these drugs have recently received considerable attention in large cardiovascular safety trials owing to favorable HF and renal benefits. For example, in patients with T2D and high CVD risk, the EMPA-REG OUTCOME trial demonstrated a 35% relative risk reduction in hospitalization for HF for empagliflozin vs placebo,^8^ and the CANVAS Program with canagliflozin demonstrated beneficial cardiovascular and renal outcomes.^9^ The CREDENCE trial, which studied the effects of canagliflozin in patients with T2D and diabetic kidney disease,^10^ reported a decrease in HF hospitalization by 39% (95% confidence interval 20%–53%), in addition to attenuating the loss of kidney function. By comparison, studies assessing the cardiovascular effects of glucose lowering per se, when mediated by other agents, had not demonstrated similar benefits, while HF outcomes may even be worsened by some glucose-lowering drugs.^11,12^

**Fig. 1.**
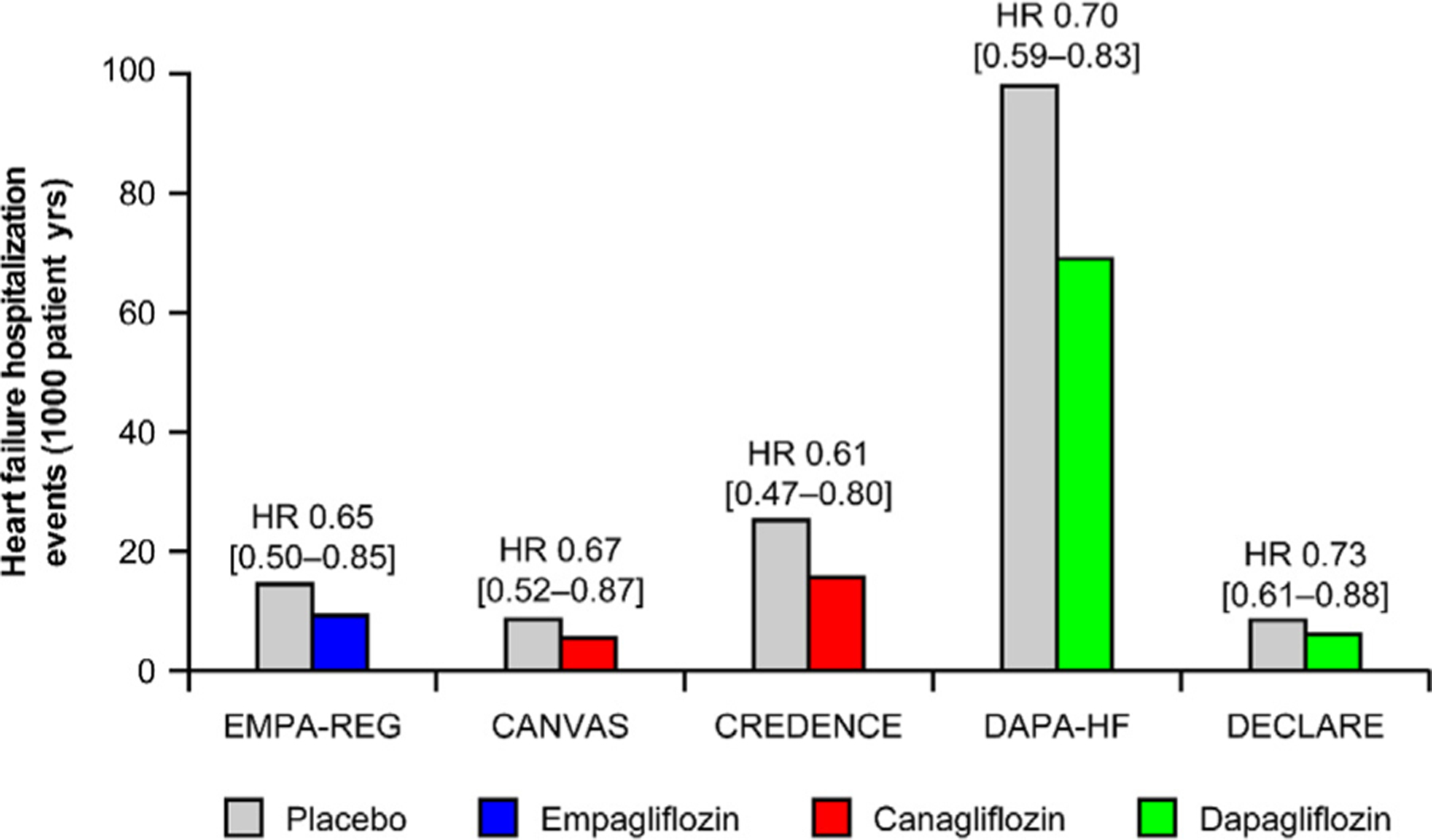
Summary of hospitalization for heart failure results from recent cardiovascular safety trials of sodium-glucose cotransporter 2 (SGLT2) inhibitors. Values in brackets are 95% confidence intervals. Values were derived from the trials’ publications; slightly different terminology was used in the trials to describe heart failure hospitalization: EMPA-REG and DECLARE used “rate per 1000 pt-yrs,” CANVAS used “number of participants per 1000 pt-yrs,” and CREDENCE and DAPA-HF used “events per 1000 [or 100 for DAPA-HF] pt-yrs.” CANVAS, CANagliflozin cardioVascular Assessment Study; CREDENCE, Canagliflozin and Renal Events in Diabetes with Established Nephropathy Clinical Evaluation; DAPA-HF, Dapagliflozin and Prevention of Adverse Outcomes in Heart Failure; DECLARE, Dapagliflozin Effect on Cardiovascular Events—Thrombolysis in Myocardial Infarction 58; EMPA-REG, EMPAgliflozin cardiovascular outcome event trial in type 2 diabetes mellitus patients—Removing Excess Glucose; HR, hazard ratio.

Since the publication of the results of these and other cardiovascular safety trials (Fig. 1), as well as real-world effectiveness studies (eg, CVD-REAL^13^), investigators and clinicians have considered a variety of potential mechanisms underlying the cardiorenal benefits of SGLT2 inhibition. It is generally agreed that (modest) decreases in blood pressure (BP), glucose concentrations, body weight, and serum urate concentrations do not fully explain the observed cardiovascular benefits.^14,15^ Although the exact pathways are not fully understood, the purpose of this article is to describe the effects of SGLT2 inhibitors on sodium handling beyond diuresis and natriuresis per se, and to discuss the proposed cardiovascular consequences of changes in sodium sensitivity and balance, including direct sodium-related cardiac effects, effects on interstitial fluid and plasma volume, and changes in nonosmotic sodium storage.

## Sodium Balance, Sodium Sensitivity, and Cardiovascular Health

Given the well-known role of sodium in cardiovascular health, we next address recent research that challenges traditional views about sodium homeostasis, with potential implications for the pathophysiology and treatment of HF, including the pathophysiological role of interstitial sodium in HF, as reviewed elsewhere.^16^ In most adult populations, the average salt intake well exceeds the approximately 5-g daily limit recommended by the World Health Organization.^17^ Excessive salt intake has been linked to hypertension, CVD,^18^ and CKD. Although the pathogenesis underlying the relationship between excessive salt intake and cardiorenal complications remains debated, the leading hypothesis for decades has been that—in so-called salt-sensitive individuals—excess sodium intake with concomitant impaired renal sodium excretion results in extracellular volume expansion and hypertension.^19^ In patients with CKD, a lower glomerular filtration rate and activation of the renin–angiotensin–aldosterone system result in an increased venous pressure, decreased renal perfusion, decreased cardiac output, and ultimately HF. The net results of these pathophysiological changes include further sodium and water retention with activation of the renin–angiotensin–aldosterone system and the sympathetic nervous system. However, carefully designed sodium-balance studies in so-called salt-resistant participants, that is, individuals in whom increased salt intake does not increase BP or body water/weight, show that much of the ingested sodium excess is in fact not excreted in the urine.^20^ Rather, these studies have proposed that sodium may be stored nonosmotically (ie, without altering the extracellular volume) at extrarenal locations, which serve to act as an osmotic sodium buffer. For example, daily rhythmic fluctuations in total body sodium content were found with large variations in 24-hour urinary sodium excretion, despite a fixed sodium intake, which suggests nonosmotic sodium accumulation and the storage of salt in a third body compartment.^20^ Osmotic excretion of significant amounts of sodium has also been shown in healthy people after hypertonic saline infusion.^21^ Using ^23^Na magnetic resonance imaging, muscle and skin were shown to contain considerable amounts of sodium without associated water retention.^22^ Another compartment that binds sodium in a nonosmotic manner and thus could influence extracellular volume and BP regulation is the endothelial surface layer, or glycocalyx, located on the luminal side of the vascular endothelium. The endothelial surface layer has abundant negatively charged glycosaminoglycans and is in direct contact with circulating blood sodium and glucose. These glycosaminoglycans have been shown to display avid sodium-binding capacity.^23^ Emerging literature has challenged the traditional 2-compartment model of sodium balance providing support to a 3-compartment model that includes the binding of sodium to glycosaminoglycans, such as those in the muscles and skin. Endothelial surface layer damage has been observed in patients with T2D^24^ and CKD,^25^ which could explain (at least in part) the salt sensitivity observed in this population. Other functions of the endothelial surface layer include the production of nitric oxide owing to shear stress, and the formation of a barrier to prevent circulating inflammatory cells from reaching underlying tissues. Consistent with these data, restoration of the endothelial surface layer by sulodexide, a mixture of endothelial surface layer constituents, has been shown to decrease BP.^26^

Although nonosmotic sodium storage seems beneficial in the short term, saturated sodium depots in the skin have been linked to both hypertension and left ventricular hypertrophy.^27^ Additionally, high vs low dietary sodium intake has been shown to increase the number of monocytes,^28^ which could trigger an inflammatory response. Furthermore, inflammation of the interstitium might drive microvascular and macrovascular stiffening and impair endothelial function.^29^

Nonosmotic buffering of sodium in tissues, such as glycocalyx, may decrease the adverse hemodynamic effects of sodium in the short term, although long-standing sodium overload may have deleterious consequences for the cardiovascular system. For example, some evidence suggests that sodium accumulation in the endothelial glycocalyx could lead to arterial stiffness.^30^ Accordingly, strategies to decrease tissue and interstitial sodium by facilitating renal sodium excretion may enhance cardiovascular health, although we stress that the concept of nonosmotic sodium storage remains theoretical; efforts are undertaken to more definetly determine its presence and role. We next discuss other mechanisms behind the putatively central role of sodium in mediating the favorable cardiorenal effects of SGLT2 inhibition.

## Effects of SGLT2 Inhibitors on Sodium Balance

**Fig. 2.**
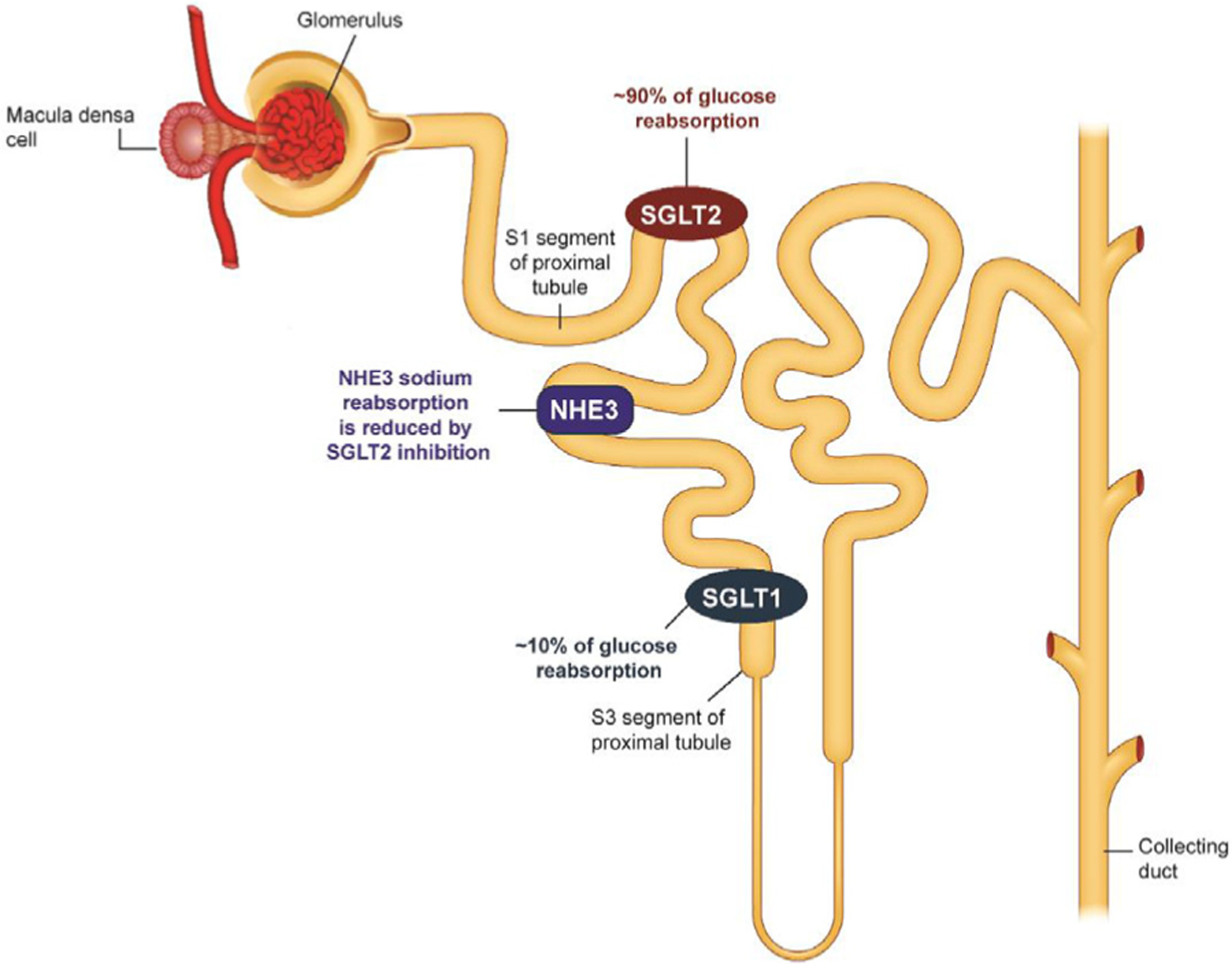
Schematic of a nephron and sodium-glucose cotransporter (SGLT) inhibitors. NHE3, Na^+^/H^+^ exchanger isoform 3; SGLT2, sodium-glucose cotransporter 2.

Although SGLT2 inhibitors were designed primarily to decrease plasma glucose concentrations, it is evident that there is concomitant inhibition of proximal tubular sodium uptake with the inhibition of glucose reabsorption. Data showing inhibition of lithium reabsorption, as a marker for proximal tubular function, support this notion.^31^ However, studies of proximal tubular sodium absorption have not been conducted in people with HF. Initial natriuresis is thought to contribute to the osmotic diuresis, which drives the increased urine output associated with acute SGLT2 inhibition, as shown in some^32,33^ but not all studies.^31^ The placebo-controlled RECEDE-CHF trial conducted in patients with T2D and HF demonstrated a significant increase in 24-hour urine volume without an increase in urinary sodium concentration when empagliflozin was used in combination with a loop diuretic.^34^ Although RECEDE-CHF did not find a significant increase in the fractional excretion of sodium with empagliflozin, another study did,^32^ a difference possibly explainable by the different time points studied between the 2 studies as well as differences in sodium intake at baseline. Notably, the major limitation of current studies showing natriuresis with SGLT2 inhibition is that study participants were not on a fixed sodium diet. The DAPASALT study, by contrast, was conducted in patients (*N* = 17) with T2D and preserved kidney function on a fixed sodium diet.^31^ The study participants received dapagliflozin and had 24-hour urine collected prior to treatment, after acute dosing, after 2 weeks of treatment, and 3 days after treatment cessation, but changes in natriuresis or plasma volume were not found.^31^ Whereas the bulk of sodium is reabsorbed in the proximal tubule, it is unclear to what extent SGLT2 transporters contribute to total sodium reabsorption in absolute terms. This phenomenon may be particularly relevant in people with T2D, in whom there is increased glucose flux through the SGLT2 transporters owing to chronic tubular hyperglycemia. It is likely that SGLT2 transporters also interact functionally with Na^+^/H^+^ exchanger isoform 3 in the proximal tubule (Fig. 2).

As such, SGLT2 inhibition is associated with marked inhibition of Na^+^/H^+^ exchanger isoform 3, even in the absence of glucose, which is likely to account for a significant proportion of the natriuresis observed with agents of this class.^35^ Another study (conducted in various animal models and human cells) did not find that SGLT2 inhibition with empagliflozin inhibited the ubiquitously expressed plasma membrane Na^+^/H^+^ exchanger Na^+^/H^+^ exchanger 1, however.^36^ Limited data exist describing proximal sodium reabsorption in humans with T2D before and during SGLT2 inhibitor therapy.^31^ The kidneys rapidly adapt to the initial natriuresis by matching sodium excretion to sodium intake, maintaining a neutral sodium balance. Therefore, sodium excretion is usually not altered with prolonged treatment,^37,38^ likely because of compensatory sodium reabsorption at more distal tubular segments. Where the additional sodium is being absorbed remains unclear. Any increase in renal sodium absorption induced by SGLT2 inhibition is likely distal to the macula densa, because the decrease in the estimated glomerular filtration rate induced by SGLT2 inhibition through tubuloglomerular feedback is thought to be driven by increased sodium and chloride concentrations detected by the macula densa. Gene expression analyses of key sodium transporters located in the distal tubule could expand knowledge of these compensatory pathways in humans.

**Fig. 3.**
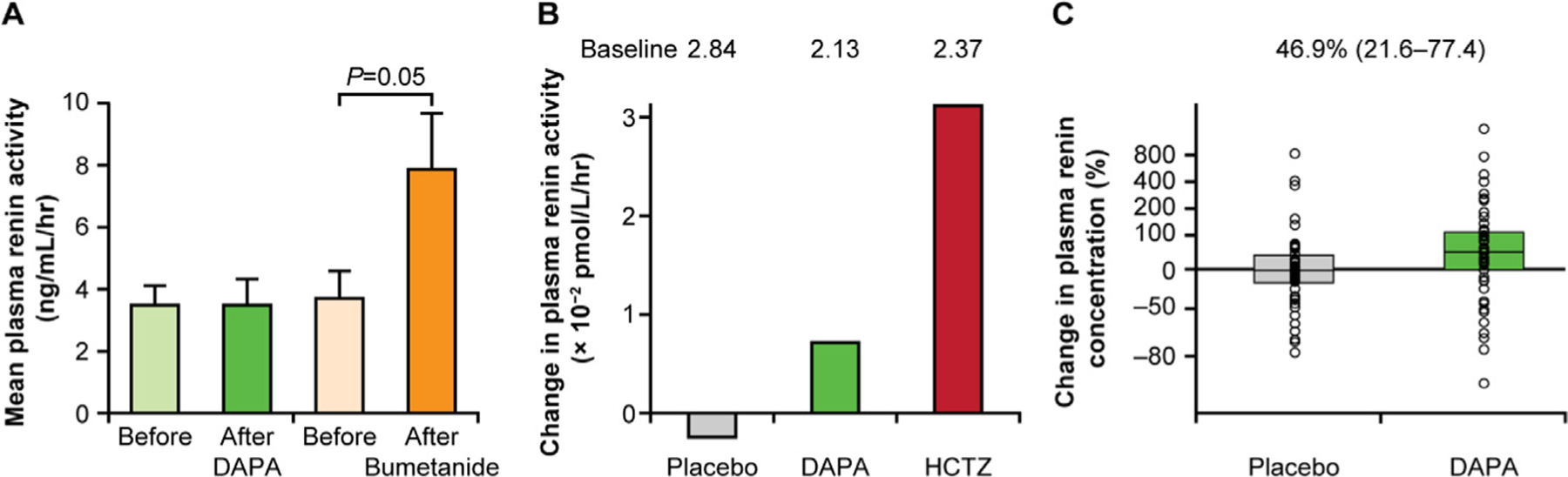
Plasma renin activity with sodium-glucose cotransporter 2 (SGLT2) inhibition. (**A**) Increase in plasma renin activity by 117% after 1 week of bumetanide treatment in healthy volunteers (adapted from Wilcox et al. 2018^41^; see Supplementary Fig. S6; available at: https://www.ahajournals.org/doi/10.1161/JAHA.117.007046; used under CC BY-NC 4.0; text slightly updated, parts of panels A and B combined, layout altered, and color added). (**B**) Week 12 change from baseline in plasma renin activity in patients with type 2 diabetes (T2D) treated with dapagliflozin (DAPA) or hydrochlorothiazide (HCTZ) (based on data from: Heerspink et al. 2013^43^, Table 2). (**C**) Changes in plasma volume markers during DAPA treatment vs placebo treatment in patients with T2D (*P* < .01) (adapted from: Eickhoff et al 2019,^50^ Fig. 2, fourth panel from the left; available at: https://www.mdpi.com/2077-0383/8/6/779; used under CC BY 4.0; text slightly updated, layout altered, and color added).

The natriuresis and osmotic diuresis associated with SGLT2 inhibition has been shown in some studies to be associated with a modest decrease in plasma volume,^39–41^ although a study of canagliflozin treatment found this decrease to be attenuated at week 12.^42^ The decrease in plasma volume is reflected by an increase in hematocrit and radioactive-labeled albumin,^43^ which is sustained during prolonged treatment but is reversed after cessation of therapy. A mathematical model-based analysis to assess the fluid effects of dapagliflozin and the loop diuretic bumetanide was recently reported, based on data acquired in a healthy volunteer study of these 2 drugs.^40,41^ A key finding of this analysis was that a similar decrease in the interstitial volume occurred in response to dapagliflozin as compared with that observed with bumetanide, but a smaller decrease was observed in plasma volume, which may result in improved tissue perfusion and less acute kidney injury incidence with SGLT2 inhibition compared with loop diuretics. Such studies highlight the differences between SGLT2 inhibitors and loop diuretics. However, the hypothesis that SGLT2 inhibitors decrease the interstitial volume is based on modeling assumptions and not on direct measurements. Further, although the mechanism by which SGLT2 inhibitors decrease the interstitial volume is not known, osmotic diuresis resulting from increased urinary glucose excretion might lead to more electrolyte-free water clearance.^32,44^ Possibly because of their different site of action in the tubular system, these drug classes have markedly different effects on potassium, uric acid, glucose, renal hemodynamics, and markers of the renin–angiotensin–aldosterone–system (Fig. 3).^40,41^ SGLT2 inhibitors have consistently been shown to modestly increase renin levels owing to their diuretic/natriuretic effect.^45^ Decreases in interstitial fluid volume may contribute to the cardiovascular benefits observed in recent cardiovascular safety trials, particularly vis-à-vis HF.^46^ A mediation analysis of the EMPA-REG OUTCOME trial found that change in hematocrit explained 51.8% of the effect of empagliflozin vs placebo on the risk of cardiovascular death.^14^ Increased hematocrit has also been observed in patients with T2D without HF^47,48^ and in patients with HF with reduced ejection fraction (HFrEF) (in which 42% had a history of diabetes at baseline).^49^

Changes in hematocrit could reflect hemodynamic changes related to plasma volume contraction, which may decrease ventricular filling pressures and cardiac workload.^14,43^ It is unclear whether the beneficial HF outcomes are partly because of a direct increase in hematocrit or attributable to factors underlying the increase in hematocrit, although the latter is more likely. Changes in hematocrit are unlikely to be explained by changes in plasma volume alone. The natriuretic response induced by SGLT2 inhibitors might restore the physiologic tubuloglomerular feedback, thus decreasing the intraglomerular pressure, as stated elsewhere in this article.^51^ Changes in kidney physiology could then lead to changes in renal oxygen metabolism that affect erythropoietin production, although this notion remains speculative. An increase in erythropoiesis, resulting from a decrease in distal tubular oxygen content secondary to increased workload, could contribute to the increase in hematocrit and could be a marker of a beneficial action of these agents, given that increased erythropoietin levels may contribute to improved myocardial oxygen delivery.^47^ It is partly through this possible sequence of events that SGLT2 inhibitors could benefit the heart. Treatment with dapagliflozin has been shown to suppress hepcidin levels.^52^ Given that SGLT2 inhibition has been shown to decrease adipose tissue inflammation in a murine model of obesity,^53^ this point also raises the intriguing possibility that SGLT2 inhibition might decrease hepcidin levels via an anti-inflammatory effect, subsequently improving anemia associated with HFrEF. The effects of SGLT2 inhibitors on interstitial fluid and circulatory volumes should be confirmed, and downstream effects explored, to better understand their cardioprotective mechanisms.

Aside from inducing changes in osmotically active sodium, SGLT2 inhibitors could also modulate nonosmotic sodium storage, although less evidence is available to support this hypothesis.^40^ In a porcine model of HF, empagliflozin decreased skin sodium content and interstitial fluid volume to a greater extent than did furosemide.^54^ In a study in which 51 participants with T2D were treated with dapagliflozin or placebo for 6 weeks, sodium content in the skin and muscles of the lower leg was measured by ^23^Na-magnetic resonance imaging.^55^ Serum sodium, 24-hour urinary sodium excretion, and muscle sodium content were not significantly changed at 6 weeks with dapagliflozin treatment; however, skin sodium content was decreased. Similar studies in patients with CKD, who commonly manifest extracellular volume overload, should be conducted.

**Fig. 4.**
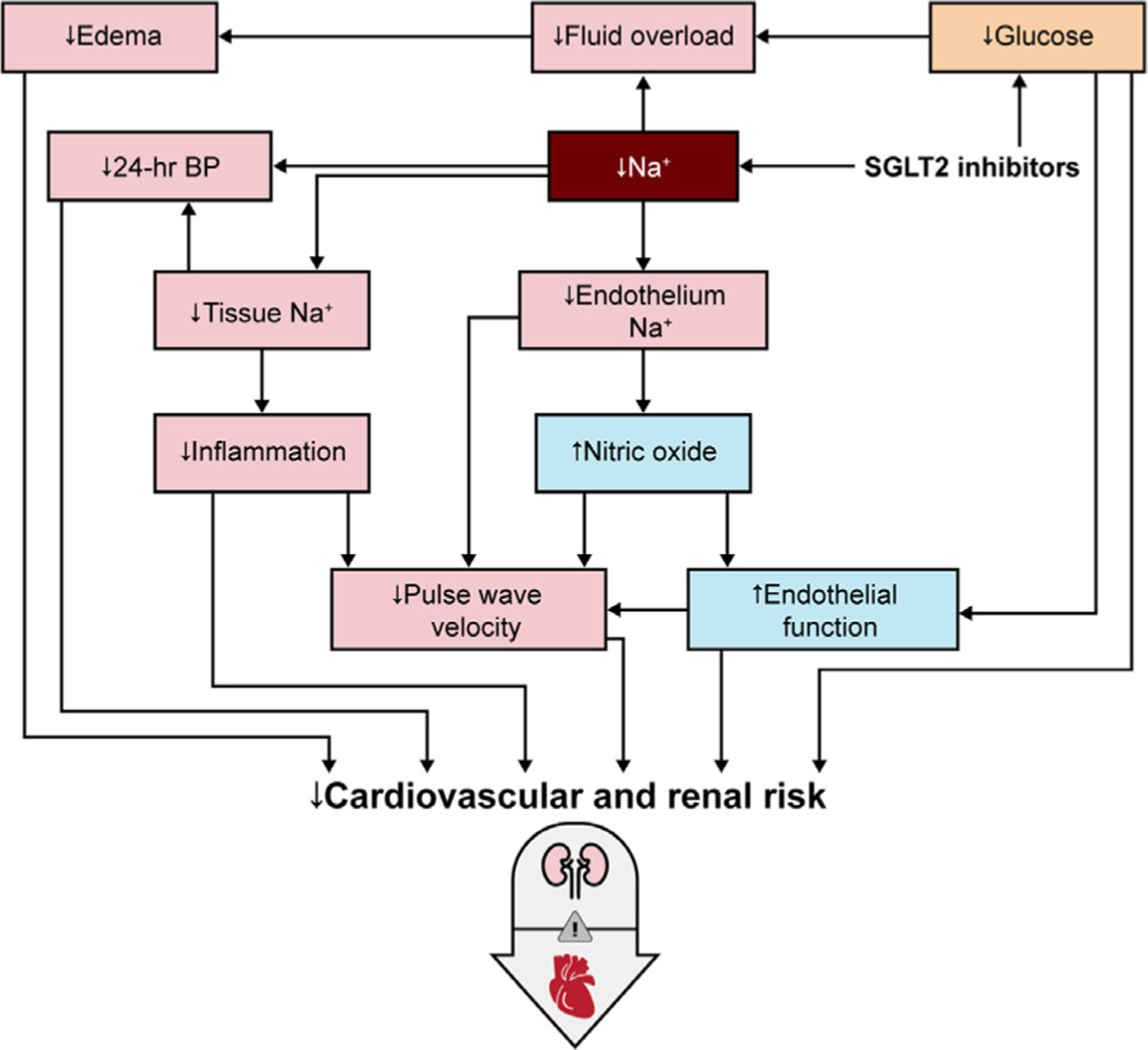
Putative sodium-centric mechanisms of benefit of sodium-glucose cotransporter 2 (SGLT2) inhibitors in people with type 2 diabetes (T2D) and cardiorenal disease. BP, blood pressure.

In summary, SGLT2 inhibitors were initially designed to reduce tubular glucose reabsorption, thereby lowering serum glucose and glycosylated hemoglobin. However, SGLT2 inhibitors might also induce a natriuretic response associated with diuresis, with a more pronounced effect on interstitial fluid compared with plasma volume. Natriuresis is unlikely to completely explain the benefits of SGLT2 inhibition, because it is transient (ie, likely present in the first 24 hours after first dosing) and modest when compared with diuretics,^33^ and recent findings may even call for a reexamination of this hypothesis.^31^ Accordingly, other consequences of SGLT2 inhibitor induced alterations in sodium handling, that is, changes in systemic hemodynamics and the vascular system, as well as potential nonosmotic sodium storage, may also contribute to the observed cardiovascular benefit (Fig. 4).

## Effects of SGLT2 Inhibition on Arterial Stiffness and Endothelial Function

SGLT2 inhibition demonstrates durable BP reduction, which may partly account for SGLT2 inhibitors’ cardiorenal benefits and may be partly driven by reductions in body sodium content.^8,9,56^ SGLT2 inhibitors have also been shown to decrease arterial stiffness and improve endothelial function. Arterial stiffness, determined largely by the elastin-to-collagen ratio in the vessel wall, is associated with the risk for cardiovascular events. Accordingly, noninvasive measures of central and peripheral arterial stiffness can serve as useful surrogate markers to determine the effectiveness of pharmacotherapies in improving cardiovascular health.^57^

Arterial stiffness, measured by pulse wave velocity, decreased in response to 8 weeks of empagliflozin 25 mg in an open-label, prospective clinical trial in young adults with type 1 diabetes mellitus.^58^ Consistent with these data, a post hoc analysis from phase III trials in adults with T2D demonstrated decreased arterial stiffness, as assessed by pulse pressure and ambulatory arterial stiffness index, and arterial resistance as measured by mean arterial pressure, in response to empagliflozin.^59^ In a pilot study of 16 adults with T2D, 2 days of dapagliflozin 12.5 mg was shown to increase flow-mediated dilatation and to decrease the pulse wave velocity and renal resistive index, independent of decreases in the BP.^60^ These data suggest that the effects of SGLT2 inhibition on systemic and renal vascular stiffness and on endothelial dysfunction are acute and persistent. Empagliflozin or dapagliflozin also restore nitric oxide production by human endothelial cells, which may contribute to the beneficial effects of SGLT2 inhibition on endothelial function, although such results were not in vivo and used high empagliflozin concentrations (1 *µ*M).^61,62^ In vivo data from a porcine model of patients with HF without diabetes indicate that empagliflozin improves nitric oxide signaling and diastolic function.^63^ A post hoc analysis of pooled data from 4 phase III studies demonstrated that canagliflozin attenuated pulse pressure and mean arterial pressure in adults with T2D.^64^ Finally, trials have also demonstrated improvement in endothelial function by reactive hyperemia peripheral arterial tonometry^65^ and flow-mediated dilatation^66^ in response to dapagliflozin in adults with T2D. The mechanisms by which SGLT2 inhibition decreases arterial stiffness and improves endothelial dysfunction are not fully understood but may be related to changes in sodium exposure.^67,68^ The effect of SGLT2 inhibition on arterial stiffness is particularly relevant to the glycocalyx, which, as noted elsewhere in this article, functions as a nonosmotic sodium buffer and can be damaged by sodium and glucose overload.

## Direct Sodium-Related Cardiac Effects of SGLT2 Inhibition

The full mechanisms of action of SGLT2 inhibition remain incompletely understood. Given the potentiating effect of increased myocardial intracellular sodium concentrations in HF, various lines of research have investigated the direct effects of SGLT2 inhibitors on the heart in in vitro model systems. Direct effects of SGLT2 inhibition on sodium concentrations in cardiomyocytes have been identified, independent of systemic effects produced via the kidney, which is remarkable given the absence of SGLT2 receptors in the heart. For example, empagliflozin was shown to decrease cardiac cytoplasmic sodium concentration via cardiac Na^+^/H^+^ exchanger inhibition.^69^ Apart from sodium-mediated effects, other direct effects, reviewed elsewhere,^70^ are possibly also involved in the long-term cardioprotective effects of SGLT2 inhibitors but are beyond the scope of the present review.

## Moving From Mechanisms to Clinical Outcomes

As briefly stated elsewhere in this article, SGLT2 inhibition has yielded remarkable effects on cardiovascular (particularly HF) and kidney outcomes in large clinical trials (Fig. 1).^8–10,56,71,72^ These results were confirmed in cardiovascular safety trials in patients with T2D and established CVD or at high risk for cardiovascular events in the DECLARE-TIMI 58^56^ and CANVAS^9^ studies, which also demonstrated improvement in HF outcomes without previous documented HF or established CVD at baseline. Because echocardiography was not performed routinely to assess the ejection fraction in these cardiovascular safety trials, any differential effect of SGLT2 inhibitors on patients with HF with preserved or reduced ejection fraction merits further study. Based on the beneficial HF findings in cardiovascular safety trials, dedicated HF trials were designed to characterize the effects of SGLT2 inhibition in preventing adverse outcomes in patients with HF. In the DEFINE-HF study, treatment with dapagliflozin indeed decreased symptoms and improved the quality of life in 263 patients with HFrEF with or without T2D, despite no significant decrease in levels of *N*-terminal pro B-type natriuretic peptide.^73^ In the DAPA-HF trial, which recruited 4744 patients with or without T2D with New York Heart Association functional class II, III, or IV HF and an ejection fraction of 40% or less, dapagliflozin decreased the primary end point of a composite of cardiovascular death or worsening HF (hospitalization or an urgent visit resulting in intravenous therapy for HF) by 26% (95% confidence interval 15%–35%).^72^ No differences were observed between individuals with or without diabetes.^72^ Confirmation of beneficial effects in patients with HFrEF has come from the EMPEROR-Reduced study, which investigated the effects of empagliflozin on HF outcomes in patients with or without diabetes.^74^ Reverse left ventricular remodeling with SGLT2 inhibition has also been demonstrated in patients with HFrEF with^75^ or without T2D.^76^

The limited effect of SGLT2 inhibitors on atherothrombotic disease (11% reduction in MACE in a recent meta-analysis^77^), and the strong effects on HF and kidney outcomes with early divergence of group outcomes (benefits seen after 3 months), strongly point to a hemodynamic effect that relates to sodium balance as postulated here. The fact that SGLT2 inhibitors improve HF outcomes to the same extent in patients with HFrEF with and without diabetes is the strongest argument to date that these beneficial effects are completely glucose independent.

Several studies that might support or refute these hypotheses are currently ongoing. The DELIVER (dapagliflozin; NCT03619213) and EMPEROR-Preserved (empagliflozin; NCT03057951) studies investigate whether the decrease in HF hospitalizations extends to patients with HF with preserved ejection fraction and with normal or near normal kidney function (without albuminuria). The phase III DAPA-CKD trial, which was stopped early based on overwhelming efficacy, reported a hazard ratio for the composite of death from cardiovascular causes or hospitalization for HF of 0.71 (95% confidence interval 0.55−0.92, *P* = .009) as well as benefit on kidney outcomes, in patients with CKD with and without diabetes.^71^ Additionally, studies such as DAPACARD (NCT03387683) and ERADICATE-HF (NCT03416270) investigate the underlying mechanisms, focusing on myocardial substrate metabolism, sodium reabsorption, and plasma volumes.

## Conclusion

This review has summarized data on the salutary effects of SGLT2 inhibition unrelated to glucose metabolism, including changes in sodium balance, sodium sensitivity, and direct sodium effects, on the heart and nonosmotic sodium stores, and hypothesized that these may partly contribute to improved HF outcomes. Importantly, however, data correlating either changes in extracellular sodium stores or direct cardiac effects with clinical outcomes are not available yet. Moreover, despite some support, the nonosmotic sodium hypothesis is novel and remains to be validated fully. A better understanding of the nonosmotic mechanisms underpinning the cardiorenal benefits of SGLT2 inhibition may allow researchers to assess the effects of SGLT2 inhibitors in combination with other drugs that affect sodium.
